# Macrophage M2 polarization induced by exosomes from adipose-derived stem cells contributes to the exosomal proangiogenic effect on mouse ischemic hindlimb

**DOI:** 10.1186/s13287-020-01669-9

**Published:** 2020-04-22

**Authors:** Dihan Zhu, Takerra K. Johnson, Yang Wang, Miracle Thomas, Ky Huynh, Qinglin Yang, Vincent C. Bond, Y. Eugene Chen, Dong Liu

**Affiliations:** 1 Cardiovascular Research Institute, Morehouse School of Medicine, 720 Westview Drive SW, Atlanta, GA 30310 USA; 2 Ophthalmic Genetics and Visual Function Branch, National Eye Institute, Bethesda, MD USA; 3 Department of Pharmacology, Louisiana State University School of Medicine, New Orleans, LA USA; 4 Department of Microbiology, Biochemistry & Immunology, Morehouse School of Medicine, Atlanta, GA USA; 5 Department of Internal Medicine, University of Michigan Medical Center, Ann Arbor, MI USA; 6 Department of Physiology, Morehouse School of Medicine, Atlanta, GA USA

**Keywords:** Exosome, Stem cells, Macrophage, Angiogenesis, microRNA

## Abstract

**Background:**

M2 macrophages and exosomes from adipose-derived stem cells (ASCs) are both reported to promote angiogenesis. However, the possible synergistic effects between exogenous exosomes and endogenous M2 macrophages are poorly understood.

**Methods:**

Exosomes were isolated from conditioned medium of normoxic and hypoxic ASCs using the combined techniques of ultrafiltration and size-exclusion chromatography and were identified with nanoparticle tracking analysis and immunoblotting for exosomal markers. Macrophages were collected from the mouse peritoneal cavity. M1 and M2 macrophages were detected by immunoblotting for the intracellular markers inducible nitric oxide synthase (iNOS) and arginase-1 (Arg-1) and by flow cytometry for the surface markers F4/80, CD86, and CD206. Murine models of Matrigel plug and hindlimb ischemia were employed as in vivo angiogenic assays.

**Results:**

When M1 macrophages were treated with exosomes from normoxic ASCs (Nor/Exo), and particularly from hypoxic ASCs (Hyp/Exo), the expression of the M1 marker iNOS decreased, and the M2 marker Arg-1 increased in a time- and dose-dependent manner. Additionally, a decrease in the M1 surface marker CD86 and an increase in the M2 surface marker CD206 were observed, which suggested that M1 macrophages were polarized to an M2-like phenotype. Conditioned medium from these M2-like macrophages presented lower levels of proinflammatory cytokines and higher levels of proangiogenic factors and promoted endothelial cell proliferation, migration, and tube formation. Furthermore, M2 polarization and angiogenesis were induced upon the administration of exosomes in mouse Matrigel plug and hindlimb ischemia (HLI) models. Interestingly, these exosomal effects were attenuated by using a colony stimulating factor 1 receptor (CSF-1R) inhibitor, BLZ945, in vitro and in vivo. Downregulation of microRNA-21 (miR-21) in hypoxic ASCs reduced the exosomal effects on M2 polarization, Akt phosphorylation, and CSF-1 secretion. A similar reduction in exosomal activity was also observed when exosomes were administered along with BLZ945.

**Conclusion:**

Our findings provide evidence that exosomes from ASCs polarize macrophages toward an M2-like phenotype, which further enhances the exosomal proangiogenic effects. Exosomal delivery of miR-21 and positive feedback of secreted CSF-1 may be involved in macrophage polarization.

## Introduction

Ischemic cardiovascular and cerebrovascular diseases continue to represent the most common cause of death in modern society. To address this concern, research on therapeutic angiogenesis has been evolving for the past decades [1]. The protein/gene approach, stem/progenitor cell approach, and subsequent microvesicle/exosome approach have been employed as therapies for ischemic diseases [2, 3]. Adipose-derived stem cells (ASCs) possess the characteristics of easy acquisition, high proliferative potential, and low immunogenicity, making them attractive candidates for cell-based therapeutics [4, 5]. Unlike living cells, exosomes are submicron vesicles that target recipient cells to deliver their cargo for cell-cell communication. Our laboratory and others have demonstrated that exosomes from ASCs have angiogenic effects [5, 6]. However, the interrelationship between the administered exosome preparations and the innate reparative aspects in angiogenesis remain largely unexplored.

Ischemic diseases, similar to infectious diseases, activate macrophages and trigger inflammatory responses to injured/infarcted tissues [7, 8]. While M1 macrophages, a classically activated subset, function in the early inflammatory stages to stimulate the inflammatory response and clean cell debris, M2 macrophages, an alternatively activated subset, accumulate in the late inflammatory stages to alleviate the inflammatory response and initiate angiogenesis and tissue repair [9–12]. In view of excess M1 macrophages in the late stages leading to exacerbated lesions and delayed recovery, it is crucial for macrophages to be polarized to the M2 phenotype on time [11]. Traditionally, M2 polarization is driven by anti-inflammatory cytokines secreted by T helper 2 cells in the late stages of infection [13]. Stem cells, including ASCs, are recently reported to induce M2 polarization through secretomes when used for tissue repair or inflammatory inhibition [14, 15]. Transfusion of mesenchymal stem cell (MSC)-educated M2 macrophages is reported to reduce tissue injury in a mouse acute kidney injury model. Severe histological and functional injury is observed if M1 macrophages are transfused [16]. Stem cell-derived exosomes have been broadly studied as a cell-free therapeutic approach. Recently, exosomes from ASCs have been implicated in reducing inflammation in white adipose tissue for anti-obesity effects by polarizing M2 macrophages [17]. In the current study, we demonstrate that in addition to promoting angiogenesis directly, exosomes from hypoxic ASCs induce M1 to M2 macrophage transition, which further enhances the exosomal proangiogenic effects in ischemic hindlimbs. Colony stimulating factor 1 (CSF-1) secretion upregulated by exosomal microRNA-21 (miR-21) delivery may mediate and have positive feedback on the transition of the macrophage phenotype.

## Materials and methods

### Cell culture

Mouse adipose-derived stem cells (ASCs) were purchased from Cyagen (Santa Clara, CA). Mouse cardiac microvascular endothelial cells (CMVECs) were purchased from Cellbiologics (Chicago, IL). Both cell types were maintained at 37 °C in a humidified 5% CO_2_ incubator. Cells at passages 4–6 were used for all experiments.

### Exosome isolation

ASCs were incubated in either normoxic or hypoxic (1% O_2_) conditions in basal medium containing 1% exosome-free FBS for 3 days. Exosomes were isolated from the incubated medium using combination techniques of ultrafiltration and size-exclusion chromatography as described previously [18]. The protein concentration of the isolated exosomes was detected by using a total exosome RNA and Protein Isolation Kit (Thermo Fisher Scientific; Waltham, MA) and a BCA Protein Assay Kit (Thermo Fisher Scientific) according to the manufacturer’s instructions. In all cases, the FBS used in this study was exosome-free FBS, which was obtained by ultracentrifugation of FBS at 100,000×*g* for 18 h at 4 °C.

### Nanoparticle tracking analysis (NTA)

An LM10 NTA device (Malvern; Amesbury, UK) was used according to the manufacturer’s recommendations. Each exosome sample was analyzed by detecting the rate of the Brownian motion of particles in liquid suspension. The analysis settings were optimized, and each video was analyzed to obtain the mean, mode, median, and estimated concentration of each particle size. A total of 500 μl of a 1:5-diluted exosome sample in phosphate-buffered saline (PBS) was injected into a NanoSight sample cubicle, which yielded a particle concentration of 1 × 10^8^ particles/ml.

### Immunoblotting

Immunoblotting was performed as previously described [6]. Proteins were detected using primary anti-CD9 (ab92726, Abcam, Cambridge, MA), anti-TSG101 (T5701, MilliporeSigma, St. Louis, MO), anti-Alix (ab186429, Abcam), anti-iNOS (13120S, Cell Signaling), anti-Arg-1 (93668S, Cell Signaling), anti-GAPDH (437,000, Thermo Fisher Scientific), anti-P (phospho)-Akt Ser473 (4060, Cell Signaling), anti-P-Akt Thr308 (13,038, Cell Signaling), and anti-Akt (4685, Cell Signaling) antibodies. Exposure of the resultant protein bands was performed with an ImageQuant LAS 4000 Luminescent Image Analyzer (GE Healthcare; Chicago, IL).

### Macrophage isolation, polarization, and polarizing inhibition

All animal experiments in this study were approved by the Institutional Animal Care and Use Committee of Atlanta University Center and complied with the NIH guidelines for the care and use of laboratory animals. Male C57BL/6J mice at 6–8 weeks of age were purchased from The Jackson Laboratory (Bar Harbor, ME). Five milliliters of PBS was intraperitoneally injected after euthanasia. Diluted peritoneal fluid was withdrawn and loaded onto a 70-μm cell strainer (Corning; Corning, NY). The filtrate was centrifuged at 250×*g* for 15 min, and the cell pellet was resuspended in Macrophage-SFM (Thermo Fisher Scientific). The cell suspension was transferred to a cell culture vessel. The attached cells were defined as M0 macrophages. M1 polarization was induced by incubating M0 macrophages in Macrophage-SFM supplemented with 10 μmol/L IFN-γ (R&D Systems, Minneapolis, MN) for 24 h and then with 10 μmol/L IFN-γ plus 10 ng/ml LPS (MilliporeSigma) for another 24 h. M2 polarization was induced by incubating M0 macrophages in Macrophage-SFM supplemented with 15 μmol/L IL-4 (R&D Systems) and 10 μmol/L IL-10 (Thermo Fisher Scientific) for 48 h [19]. M2 macrophages were used as a positive control in this study. BLZ945 (Selleck Chemicals, Houston, TX), an M2 polarization inhibitor that inhibits colony stimulating factor 1 receptor (CSF-1R) [20, 21], was used at a concentration of 30 ng/ml along with exosomes when indicated.

### Exosome uptake

Exosomes from ASCs were labeled using an ExoGlow-Protein EV Labeling Kit (System Biosciences; Palo Alto, CA) according to the manufacturer’s instructions. M1 macrophages were incubated in the labeled exosome suspension at a concentration of 30 μg/ml in Macrophage-SFM for the indicated times. The cells were then counterstained with Hoechst 33342 (Thermo Fisher Scientific) and examined by fluorescence microscopy (Olympus, Japan) and flow cytometry [18].

### Flow cytometry analysis of macrophage markers

M1 macrophages were incubated with 30 μg/ml exosomes for 48 h. The cells were harvested and blocked with 3% BSA in PBS for 30 min, followed by incubation with FITC-anti-F4/80 and PE-anti-CD86 or PE-anti-CD206 antibodies (BioLegend; San Diego, CA) in the dark. The cells were then analyzed by flow cytometry. The ExpressPlus program was selected. The cells incubated with FITC-isotype and PE-isotype (BioLegend) were used to exclude nonspecific binding by the fluorescent antibodies. M1 macrophages were defined as F4/80^+^ and CD86^+^. M2 macrophages were defined as F4/80^+^ and CD206^+^.

### ELISA

M1 macrophages were incubated with 30 μg/ml exosomes for 48 h. The cells were then washed and incubated in fresh Macrophage-SFM for another 48 h. The culture medium was collected and centrifuged at 500×*g* for 10 min at 4 °C to remove unadhered cells. The supernatant was used as conditioned medium (CdM). The concentrations of the cytokines and growth factors interleukin-4 (IL-4), IL-6, macrophage-CSF (M-CSF), granulocyte-macrophage-CSF (GM-CSF), vascular endothelial growth factor (VEGF), and basic fibroblast growth factor (bFGF) in the CdM were determined with corresponding ELISA kits (R&D Systems) according to the manufacturer’s instructions.

### Cell proliferation

CMVECs were quiesced with endothelial basal medium/1% FBS for 24 h and incubated in a 5 μmol/L fluorescent carboxyfluorescein succinimidyl ester (CFSE) solution (green) from the CellTrace CFSE Cell Proliferation Kit (Thermo Fisher Scientific) at 37 °C for 20 min. The cells were then washed and incubated in each of the various CdMs from macrophages as described above, buffered with an equal volume of endothelial basal medium/1% FBS. The same medium was changed on day 2. The cells were harvested at day 4 and subjected to analysis using a flow cytometer with 488-nm excitation and emission filters appropriate for fluorescein.

### Migration assay

CMVECs were incubated in each of the various CdMs from macrophages as described above, buffered with an equal volume of endothelial basal medium/1% FBS in a 24-well insert plate (BD Biosciences; San Jose, CA) at 37 °C for 24 h according to the manufacturer’s instructions. Cell nuclei were stained with Hoechst 33342. The cells that migrated to the lower side of the insert were counted under a fluorescence microscope as previously described [22].

### Tube formation assay

CMVECs were seeded on plates precoated with growth factor-reduced Matrigel (BD Biosciences) and incubated in each of the various CdMs buffered with an equal volume of endothelial basal medium/1% FBS as described above for 4 h. The cells were then stained with Calcein AM (Thermo Fisher Scientific), and tube formation was visualized using fluorescence microscopy. Total vessel length, vessel area, number of junctions, and network complexity were calculated using AngioTool v.2 software [18].

### Matrigel plug assay

Ice-cold Matrigel containing 100 μg of exosomes from normoxic adipose-derived stem cells (Nor/Exo) or exosomes from hypoxic adipose-derived stem cells (Hyp/Exo) at a volume of 250 μl per plug was subcutaneously injected into the flanks of male, 6–8-week-old C57BL/6J mice (The Jackson Laboratory) as previously described [6]. The plugs were collected 2 weeks after implantation. One half of each plug was used for immunohistochemistry analysis, and the other half was digested with 2 mg/ml collagenase type I in PBS containing 20 mM HEPES at 37 °C for 30 min and filtered to obtain the cell suspension for flow cytometry analysis as described above.

### Hindlimb ischemia model

The femoral artery was ligated on the left hindlimb of male C57BL/6J mice (The Jackson Laboratory) at 6–8 weeks of age as reported [23]. The mice were subjected to intramuscular injection in the left adductor muscle [24] with 30 μg of Nor/Exo or Hyp/Exo in 30 μl of PBS immediately after ligation. The same intramuscular injections were performed at the surgery site 4 and 8 days post surgery. Blood perfusion on the plantar surface of the hind paws was measured immediately before and after surgery and 3, 7, 14, and 21 days post surgery using laser speckle imaging (LSI, Perimed AB, Järfälla, Sweden). The left to right ratio (L/R) was used to represent the relative blood perfusion rate at the left hindlimb for each animal (*n* = 8). The mice were euthanized 3 weeks after the surgery. The left gastrocnemius muscle was collected for immunohistochemical analysis as described below. The left adductor muscle was also collected. One half was used for immunohistochemical analysis. The other half was cut into tiny pieces (~ 1 mm^3^) and digested with 3 mg/ml collagenase type I in PBS containing 20 mM HEPES at 37 °C for 40 min. The suspension was passed through a 70-μm cell strainer (Corning). The filtered cells were used for flow cytometry analysis.

### Immunohistochemistry

The cryosections of Matrigel plugs were stained with anti-CD31 antibody (AF3628, R&D Systems) as previously described [6]. The muscle specimen samples were fixed, paraffin embedded, and sectioned. The sections were incubated with the primary anti-α-smooth muscle actin (α-SMA, 19245, Cell Signaling) or anti-CD31 antibody (550,274, BD Biosciences) and counterstained with Hoechst 33342. The regions containing the most intense CD31^+^ areas of neovascularization were chosen for quantification. Five hotspots per section and 3 sections per plug or muscle specimen were analyzed at × 400 magnification. Image-Pro Plus software (Media Cybernetics) was used to measure CD31^+^ areas in each hotspot. Arteriole density was assessed as α-SMA^+^ vessels/mm^2^.

### RNA isolation and microRNA (miRNA) profiling

Total RNA was extracted from cells or exosomes using TRIzol Reagent (Thermo Fisher Scientific) according to the manufacturer’s instructions. The miRNA profiling of extracted RNA was performed by LC Sciences (Houston, TX) (*n* = 2) using a microfluidic chip.

### RT-PCR for miRNAs

Reverse transcription and quantitative real-time PCR were performed using the TaqMan miRNA assay system (Thermo Fisher Scientific) according to the manufacturer’s instructions and our previous description [6]. The relative miRNA levels were normalized to endogenous U6 small nuclear RNA levels for each sample.

### Transduction of recombinant lentivirus

The procedures performed here followed the National Institutes of Health guidelines for recombinant DNA research. All recombinant lentiviruses used in this study were purchased from Amsbio (Abingdon, UK). For transduction, ASCs were incubated with Lenti/ZipmiR-21 (ZipmiR-21) or Lenti/ZipmiR-Control (ZipmiR-Cont) at an MOI of 2 in the presence of 8 μg/ml polybrene for 48 h before further treatment.

### Statistical analysis

Statistical significance between the two groups was evaluated with a two-tailed Student’s *t* test. For multiple group comparisons (Matrigel plug assay and hindlimb ischemic model), one-way ANOVA followed by Tukey’s multiple comparison test was performed. All values are reported as the mean ± SD. Significance was accepted at the level of *p* < 0.05.

## Results

### Exosomes from ASCs, particularly from hypoxic ASCs, polarize M1 macrophages to the M2-like phenotype

**Fig. 1:**
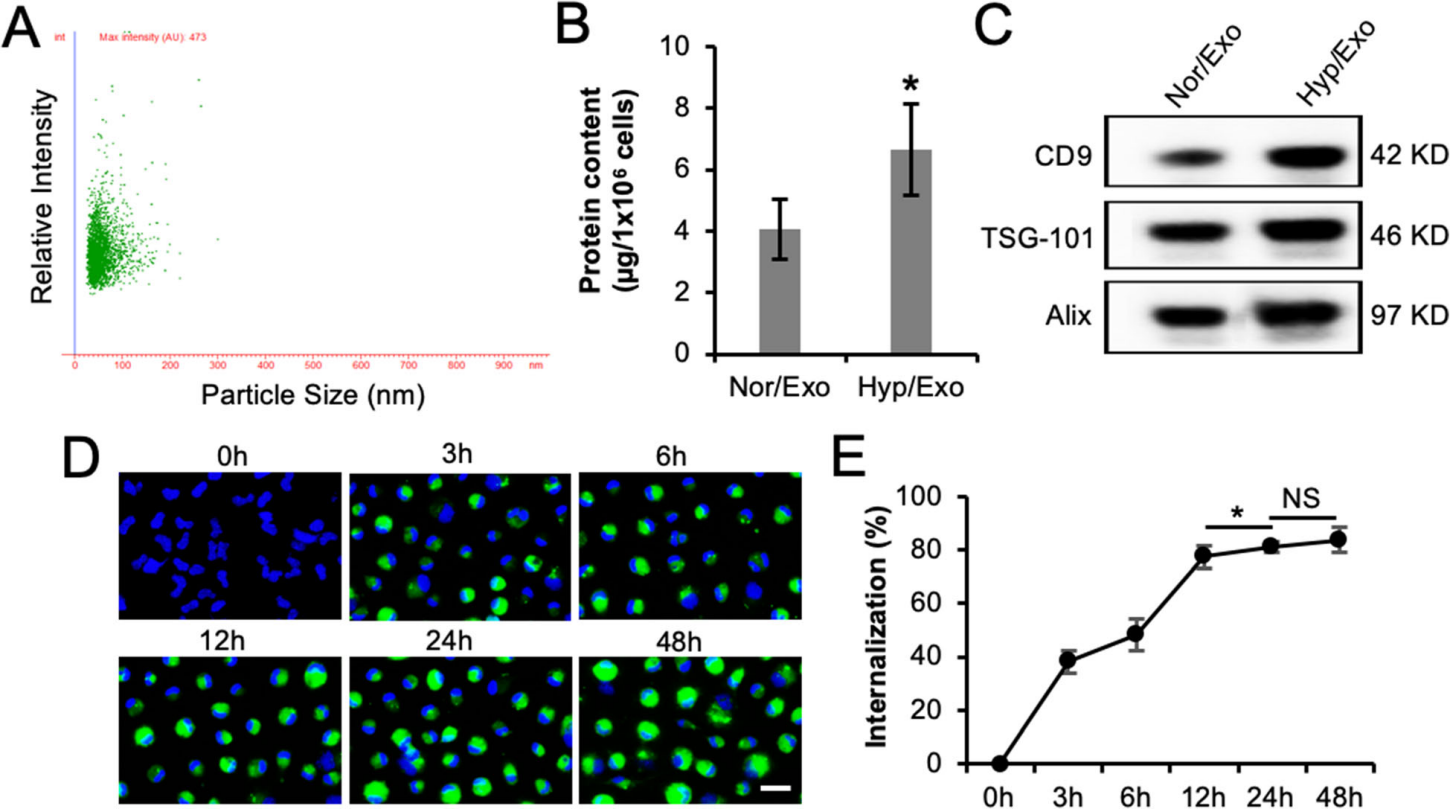
Characterization of exosomes from ASCs. **a** The isolated exosomes were examined using NTA. Scatter plot graphs of exosomes demonstrated the particle size versus light intensity of exosomes (*n* = 2). **b** Protein content of exosomes from the same number of normoxia- or hypoxia-preconditioned ASCs was compared (*n* = 5). **c** The exosomal markers CD9, TSG101, and Alix in exosomes from ASCs were determined by immunoblotting analysis. Each lane represents an exosomal lysate collected from 1 × 10^6^ ASCs (*n* = 4). **d**, **e** Uptake of ASC-derived exosomes by M1 macrophages. M1 macrophages were incubated with Exo-Green-labeled exosomes at a concentration of 30 μg/ml for the indicated times (*n* = 3). Merged images display Exo-Green (green) for labeled exosomes and Hoechst 33342 (blue) for nuclei (scale bar, 100 μm) (**d**). The percentage of exosomal internalization by M1 macrophages was measured using flow cytometry (**e**). *p < 0.05. NS, not significant

Exosomes are microparticles secreted from cells in sizes ranging from 30 to 150 nm [25]. In this study, NTA was used to determine the size distribution profile and relative particle density. The Hyp/Exo samples were within the expected range (Fig. 1a), and the Nor/Exo sample displayed a similar size distribution (data not shown). The amount of protein was used for exosome quantification. The higher protein amount (Fig. 1b) and higher levels of the exosomal markers CD9, TSG-101, and Alix (Fig. 1c) from the same number of hypoxic ASCs than normoxic ASCs suggested that ASCs secreted more exosomes upon hypoxia stimulation. To examine the polarization effect of the exosomes on M1 macrophages, M0 macrophages were collected from the peritoneal cavity of wild-type mice and subsequently polarized to M1 macrophages with standard cytokine treatment [19]. A typical pattern of the M1 marker iNOS and M2 marker Arg-1 determined by immunoblotting demonstrated the successful establishment of primary M1 macrophages along with the M2 macrophages used as a control in the following experiments (Figure I in the online-only Data Supplement). A time course experiment of exosome uptake by M1 macrophages was performed to investigate the uptake efficiency. An increase in Hyp/Exo internalization into M1 macrophages with prolonged incubation time was observed in fluorescent images (Fig. 1d). The flow cytometry analysis revealed that the percentage of uptake gradually increased up to 85% in 48 h (Figure II in the online-only Data Supplement and Fig. 1e). The uptake of Nor/Exo by M1 macrophages was similar to that of Hyp/Exo (data not shown).

**Fig. 2:**
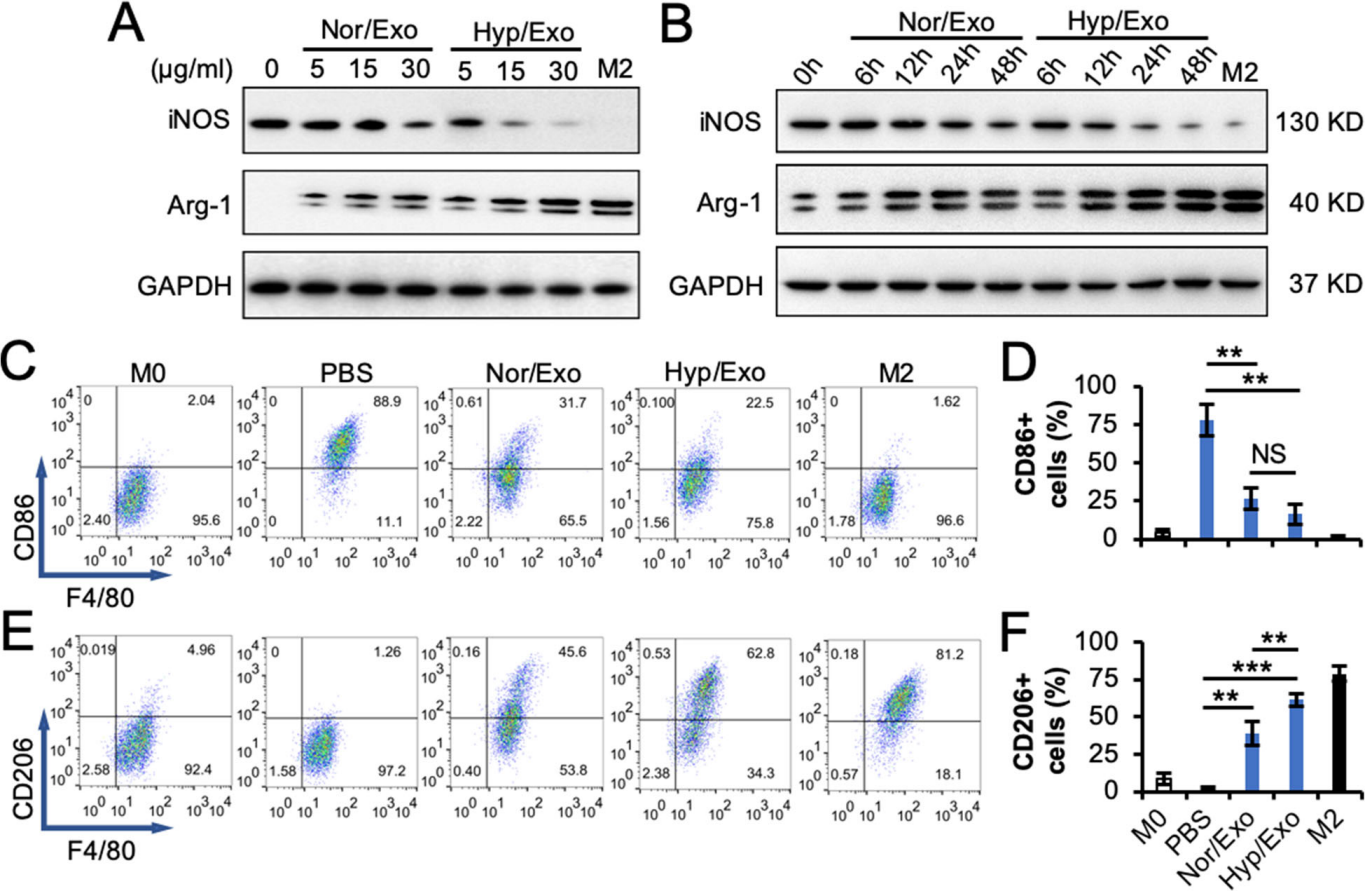
M1 macrophages are polarized to an M2-like phenotype by exosomes from ASCs. **a**, **b** Macrophage intracellular markers, iNOS for M1 and Arg-1 for M2, in M1 that were treated with the indicated concentration of Nor/Exo or Hyp/Exo for 48 h (**a**) or treated with 30 μg/ml Nor/Exo or Hyp/Exo for the indicated times (**b**) were examined by using immunoblotting. GAPDH was used as a loading control (*n* = 3). **c**–**f** Macrophage surface markers, CD86 (**c**) for M1 and CD206 (**e**) for M2, on M1 that were treated with 30 μg/ml Nor/Exo or Hyp/Exo for 48 h were examined by using flow cytometry (*n* = 3). Panels **d** and **f** are the statistical results of panels **e** and **g**, respectively. F4/80 was used as a pan-macrophage marker. M0 was used as a negative control. M2 was used as a positive control. **p < 0.01 and ***p < 0.001. NS, not significant

Upon the treatment of M1 macrophages with Nor/Exo or Hyp/Exo, iNOS was downregulated in a dose- (Fig. 2a) and time- (Fig. 2b) dependent manner, particularly when the cells were treated with Hyp/Exo. The opposite phenomenon was observed for Arg-1 when M1 macrophages were subjected to the same treatment. In addition, upon treatment of M1 macrophages with Nor/Exo or Hyp/Exo, the M1 surface marker CD86 was downregulated, while the M2 surface marker CD206 was upregulated (Fig. 2c–f). Hyp/Exo showed a stronger impact on the changes in surface markers compared with Nor/Exo. These data suggest that exosomes from ASCs promote the polarization of M1 macrophages into M2-like macrophages.

To investigate whether a similar polarization effect exists in human cells, human M1 macrophages were treated with Hyp/Exo from human ASCs. Flow cytometry analysis showed that CD80 (human M1 surface marker)-positive cells were decreased, while CD163 (human M2 surface marker)-positive cells were increased (Figure IIIA and IIIB in the online-only Data Supplement). Immunoblotting analysis showed that STAT1 (human M1 intracellular marker) was decreased and STAT3 (human M2 intracellular marker) was increased (Figure IIIC in the online-only Data Supplement). These observations suggest that human M1 macrophages could also be polarized to M2-like macrophages by exosomes from human ASCs.

### Proangiogenic effects of exosome-induced M2-like macrophages in vitro

**Fig. 3:**
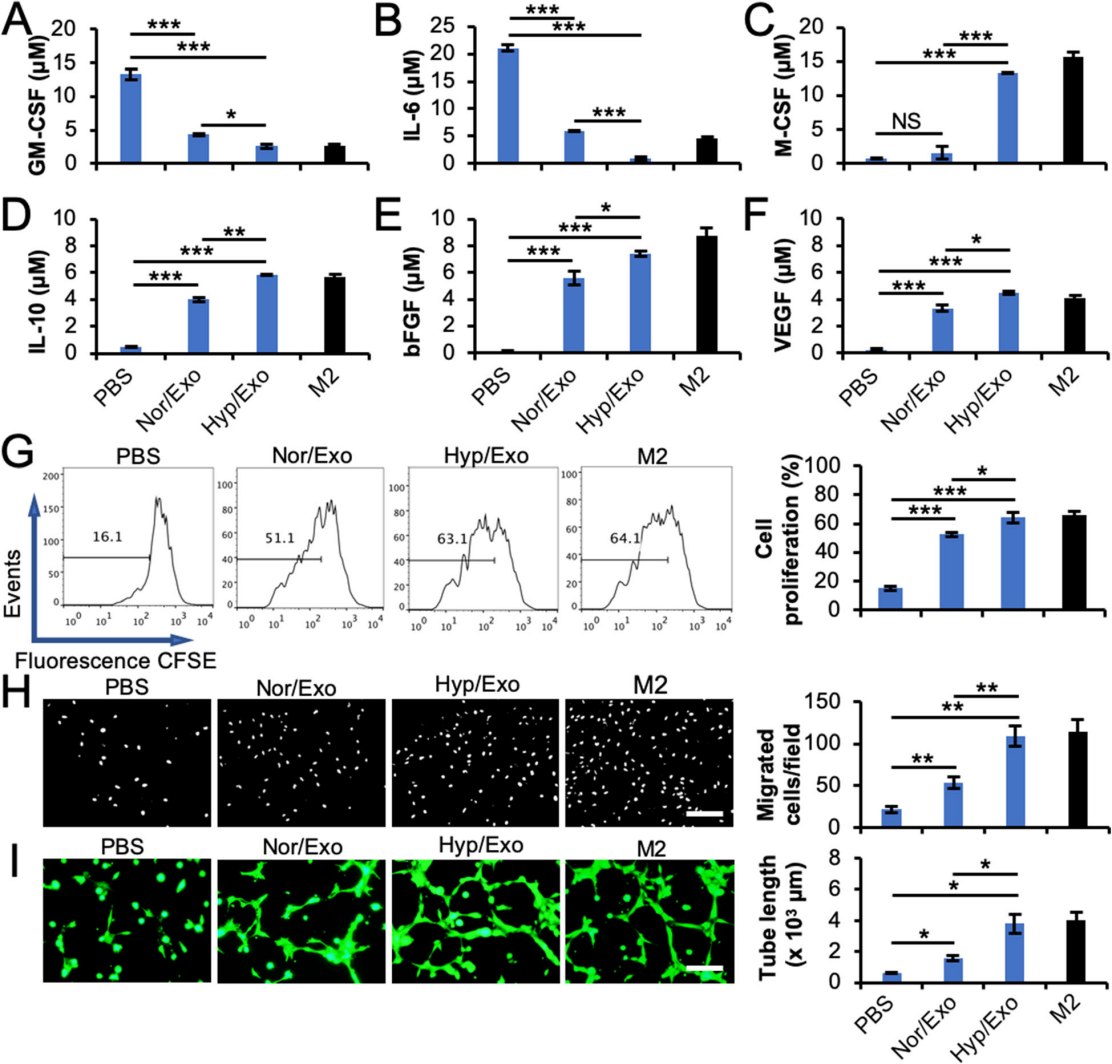
The factors secreted by M1 macrophages upon treatment with exosomes from ASCs become M2-like. **a**–**f** M1 macrophages were incubated with 30 μg/ml Nor/Exo or Hyp/Exo for 48 h. The cells were then washed and incubated in fresh Macrophage-SFM for another 48 h. The culture medium was collected as conditioned medium (CdM). The concentrations of M1-associated cytokines, GM-CSF (**a**) and IL-6 (**b**); M2-associated cytokines, M-CSF (**c**) and IL-10 (**d**); and M2-associated growth factors, VEGF (**e**) and bFGF (**f**), in CdM were measured using ELISA. M2 was used as a positive control. **g**–**i** CdM from exosome-polarized M2-like macrophages promotes angiogenesis in CMVECs. M1 macrophages were incubated with 30 μg/ml Nor/Exo or Hyp/Exo for 48 h. The cells were then washed and incubated in fresh Macrophage-SFM for another 48 h. The culture medium was collected and used as CdM. CMVECs were treated with various CdMs from macrophages. Cell proliferation (**g**), migration (**h**), and tube formation (**i**) assays of CMVECs were performed as described in the “Materials and methods” section. CdM from M2 was used as a positive control. *p < 0.05, **p < 0.01, and ***p < 0.001. All data are expressed as the mean ± SD of *n* = 3

It has been reported that M1 macrophages secrete proinflammatory factors, such as GM-CSF, IL-6, TNF, IL-1b, and IL-17, while M2 macrophages secrete anti-inflammatory and proangiogenic factors, such as M-CSF, IL-10, IL-4, TGF-β, bFGF, and VEGF [11, 19, 26]. To investigate the cytokines and growth factors secreted by our exosome-induced M2-like macrophages, CdM was collected from these cells and subjected to ELISA analysis. The results demonstrated that the concentrations of the cytokines GM-CSF (Fig. 3a) and IL-6 (Fig. 3b) were decreased, while M-CSF (Fig. 3c) and IL-10 (Fig. 3d) as well as the growth factors bFGF (Fig. 3e) and VEGF (Fig. 3f) were increased in CdM from M1 macrophages upon treatment with Nor/Exo or Hyp/Exo. These data confirm the impact of exosomes from ASCs on M2 polarization and imply an angiogenic function of polarized M2-like macrophages. To investigate the angiogenic effect, CdM was therefore used to treat CMVECs. The results showed that CdM from Nor/Exo- or Hyp/Exo-treated M1 macrophages promoted CMVEC proliferation (Fig. 3g), migration (Fig. 3h) and tube formation (Fig. 3i and Figure IVA through IVC in the online-only Data Supplement). Our findings provide evidence that Nor/Exo, particularly Hyp/Exo, induced M2-like macrophages to secrete more angiogenic cytokines and growth factors as well as to promote CMVEC angiogenesis in vitro.

### Exosome-induced angiogenesis and M2 polarization in mouse Matrigel plug and HLI models

**Fig. 4:**
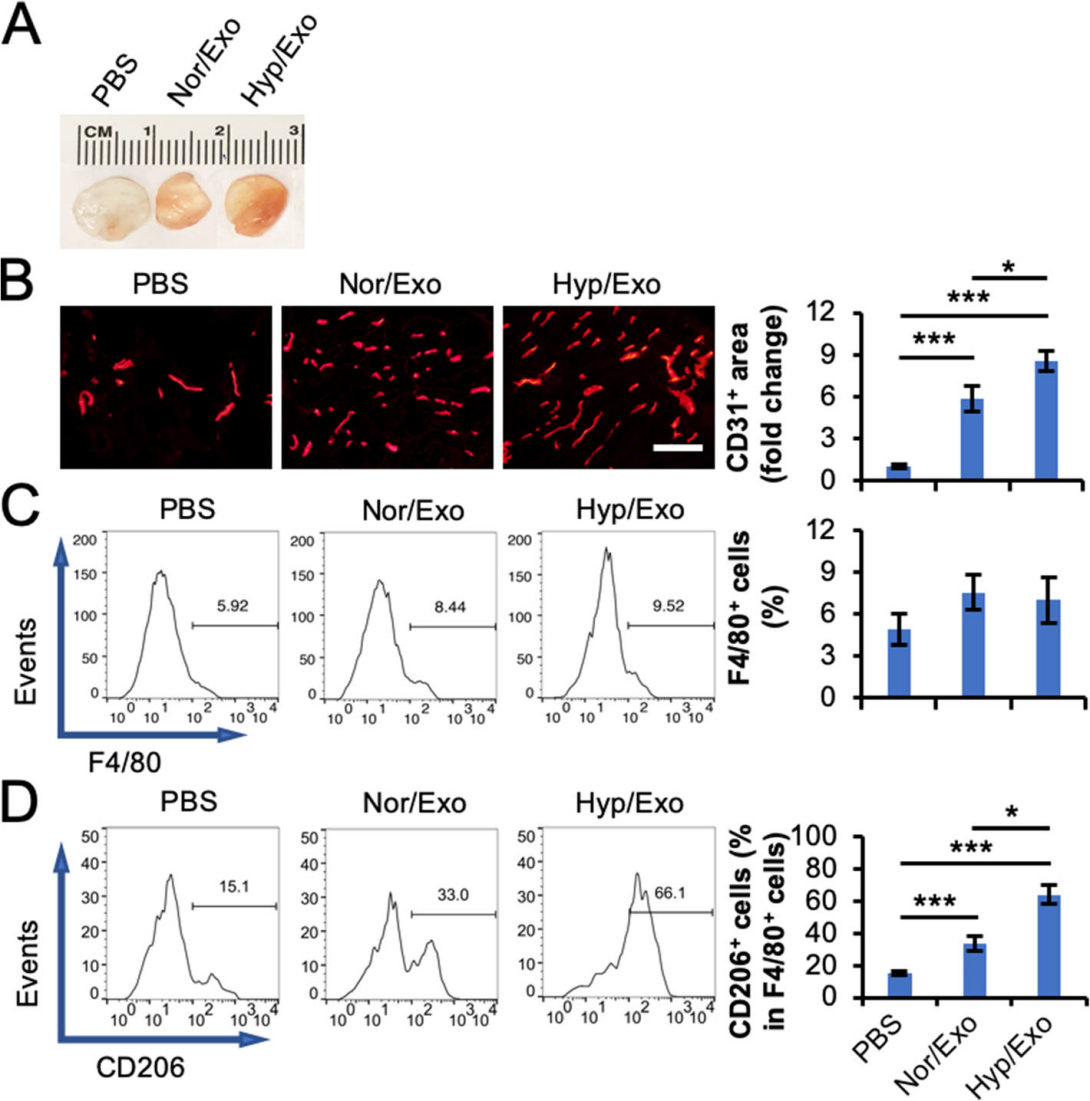
Exosomes from ASCs promote angiogenesis in a mouse Matrigel plug assay. Matrigel containing Nor/Exo or Hyp/Exo was subcutaneously injected into the flanks of mice (*n* = 6). The Matrigel plugs were harvested 2 weeks post implantation. **a** Representative images of the plugs are shown. **b** The sections of the plugs were subjected to immunohistochemistry analysis for CD31, an endothelial cell marker. Scale bar = 100 μm. Quantification of the CD31^+^ area was performed. The positive area in the slide with plugs containing PBS was set to 1. **c**, **d** Cells collected from the plugs were subjected to flow cytometry analysis. The percentage of F4/80^+^ cells in the total analyzed cells is displayed (**c**). The percentage of CD206^+^, an M2 macrophage marker, cells in F4/80^+^ cells was further analyzed (**d**). *p < 0.05 and **p < 0.01

To study the role of exosomes from ASCs in angiogenesis and M2 polarization under pathophysiological conditions, a mouse Matrigel plug assay was conducted. Our results revealed that Nor/Exo or Hyp/Exo enhanced functional vasculature formation, as indicated by the red appearance of the plugs (Fig. 4a) and the CD31-positive area in the plug section (Fig. 4b). The number of pan macrophages which defined by F4/80-positive was not affected by either Nor/Exo or Hyp/Exo (Fig. 4c). However, the percentage of CD206-positive cells in F4/80-positive cells was increased in the plugs containing Nor/Exo or Hyp/Exo (Fig. 4d). These results provide evidence that ASC-derived exosomes, especially hypoxia-preconditioned ASC-derived exosomes, promote angiogenesis and increase the proportion of M2 macrophages in the plug.

**Fig. 5:**
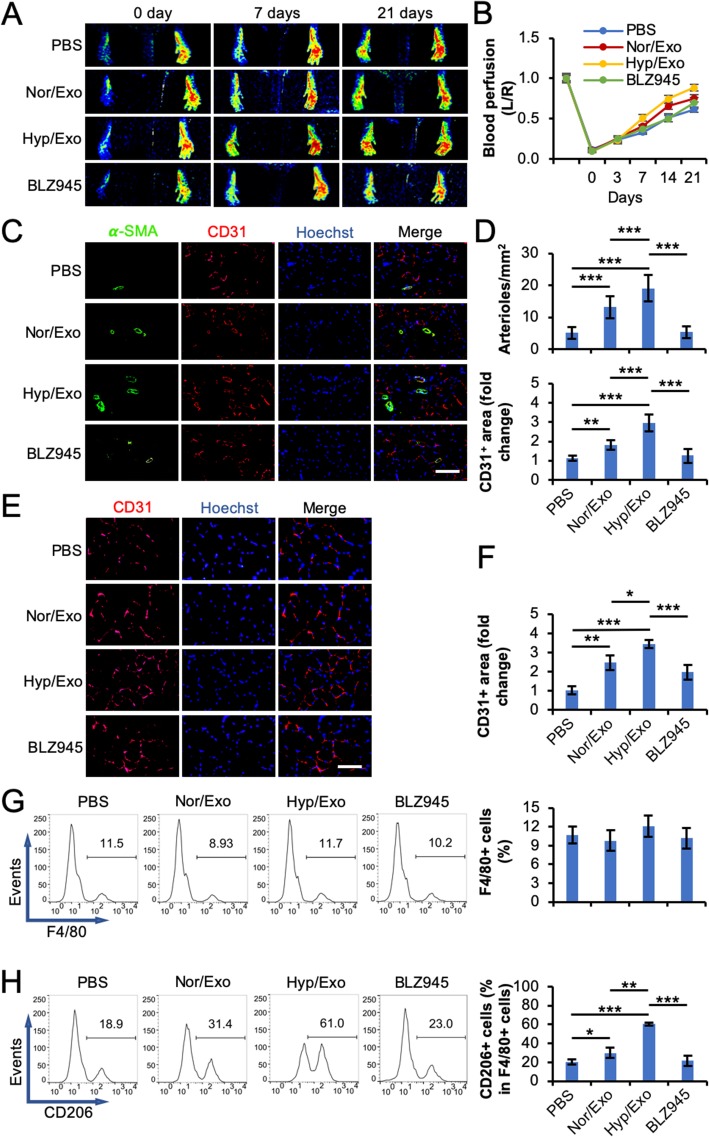
Exosomes from ASCs promote angiogenesis and M2 polarization in a mouse HLI model. After left femoral artery ligation, PBS, Nor/Exo, Hyp/Exo, or Hyp/Exo plus BLZ945 (BLZ945), an M2 polarization inhibitor that targets CSF-1R, was intramuscularly injected (*n* = 8). **a** Representative laser speckle images showing the different recovery of blood perfusion in the hind paws on the indicated days. **b** Quantitative analyses of the images showing the left/right ratio of plantar perfusion. **c**–**f**, The mice were euthanized 3 weeks post surgery. The sections of the adductor muscle from the ligated side were subjected to immunohistochemistry analysis for α-SMA, a smooth muscle marker, and CD31, an endothelial cell marker, and counterstained with Hoechst 33342 (**c**, scale bar 150 μm). Quantification of the arteriole number per square millimeter and the CD31^+^ area (**d**). The sections of the gastrocnemius muscle from the ligated side were subjected to immunohistochemistry analysis for CD31 and counterstained with Hoechst 33342 (**e**, scale bar 100 μm). Quantification of the CD31^+^ area (**f**). The CD31^+^ area on the slide from the mouse administered PBS was set to 1. **g** Flow cytometric analysis of the percentage of F4/80^+^ cells in cells dissociated from the adductor muscle. **h** The percentage of CD206^+^ cells in the F4/80^+^ cell population. GAPDH was used as a loading control. *p < 0.05, **p < 0.01, and ***p < 0.001

A mouse hindlimb ischemia (HLI) model was also used to explore the effects of exosomes on angiogenesis and macrophage polarization [23]. The results demonstrated that blood perfusion in the hind paw was enhanced by the administration of Nor/Exo starting from day 14 (*p* < 0.05) or Hyp/Exo starting from day 7 (*p* < 0.05) post femoral artery ligation in comparison with perfusion with the administration of PBS (Fig. 5a and b). The results of immunohistochemistry analysis showed that the vascular smooth muscle cell marker α-SMA was elevated by Nor/Exo, particularly by Hyp/Exo in the adductor muscle (Fig. 5c and d). Similar increases were observed for the vascular endothelial cell marker CD31 in the adductor muscle (Fig. 5c and d) and the gastrocnemius muscle (Fig. 5e and f). Interestingly, the administration of BLZ945, which is reported to block M2 polarization by inhibiting the activation of CSF-1R [20, 21], was observed to decrease Hyp/Exo-upregulated expression of α-SMA and CD31. To explore the phenotype of macrophages at the ligation site, the macrophages in the adductor muscle were analyzed by flow cytometry. The data showed that upon treatment with either Nor/Exo or Hyp/Exo, the percentage of F4/80-positive macrophages in the total analyzed cells from the adductor muscle was unchanged (Fig. 5g). However, the percentage of CD206-positive cells in the F4/80-positive macrophages was increased upon the administration of Nor/Exo or Hyp/Exo (Fig. 5h). Similar to the in vitro observation above, BLZ945 impaired the polarizing effect of Hyp/Exo on macrophages in the adductor muscle. These data suggest that Nor/Exo or Hyp/Exo from ASCs promote arteriogenesis and angiogenesis, which may be partially through CSF-1R-induced M2 polarization (but not macrophage recruitment) in this animal model.

### Exosomes from hypoxic ASCs induce M2 polarization via transport of miR-21

**Fig. 6:**
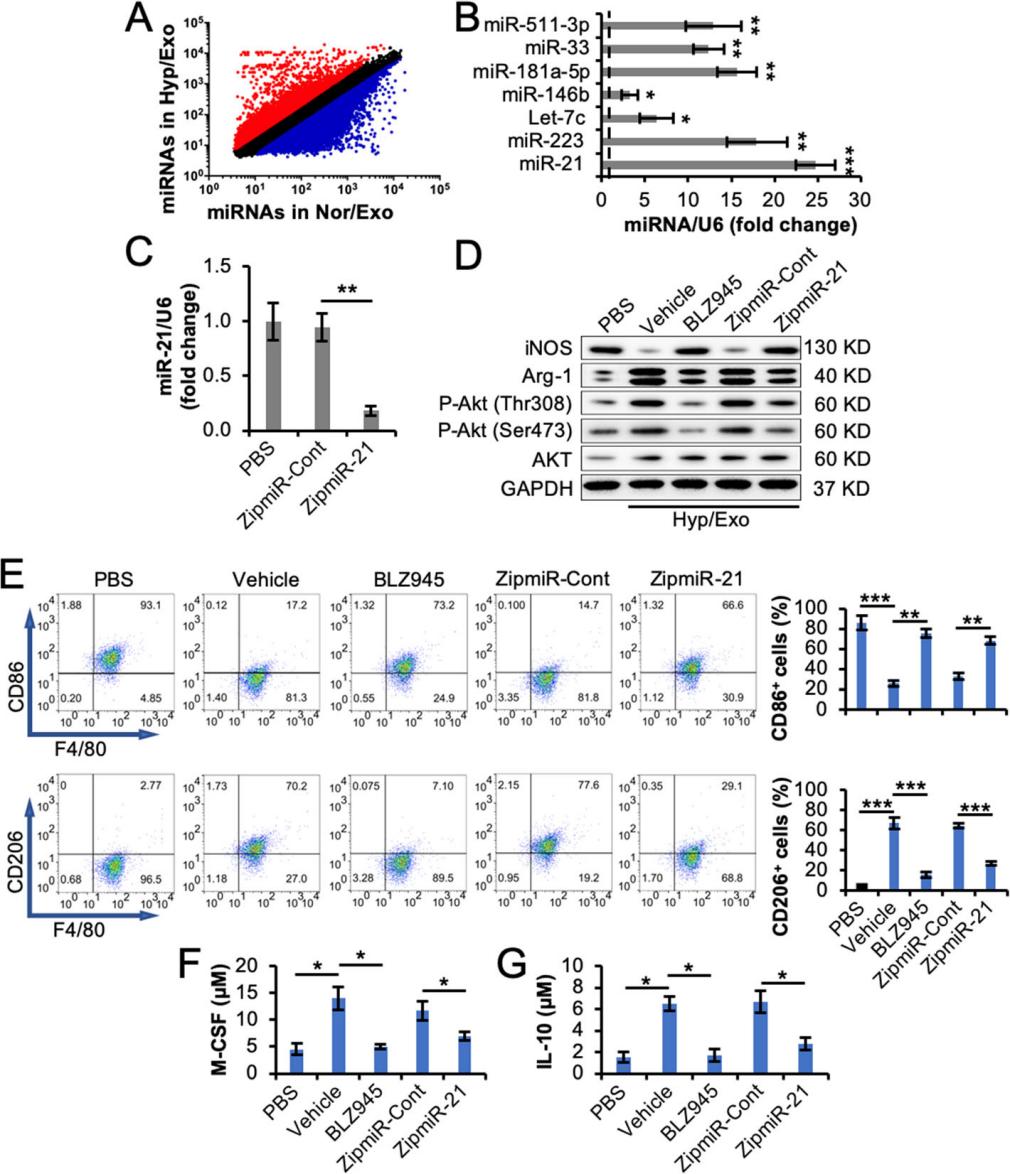
miR-21 enriched in Hyp/Exo from ASCs contributed to M2 polarization. **a** Profiling of miRNAs in Nor/Exo and Hyp/Exo was performed (*n* = 2). The relative average levels of each miRNA in Nor/Exo and Hyp/Exo are shown. Each dot represents one miRNA. The miRNAs with a ratio of Hyp/Exo to Nor/Exo greater than 2 are marked red, and those less than 0.5 are marked blue. All the other miRNAs in between are marked black. **b** The levels of miRNAs that satisfy both marked red in panel (**a**) and reported to promote M2 polarization were further examined with RT-PCR analysis. **c** Downregulation of miR-21 in Hyp/Exo from ASCs. Hypoxic ASCs were untransduced (PBS) or transduced with Lenti/ZipmiR-Cont (ZipmiR-Cont) or with Lenti/ZipmiR-21 (ZipmiR-21) to knockdown miR-21. Hyp/Exo secreted from these cells were isolated. The level of miR-21 in the different Hyp/Exo groups was examined with RT-PCR to verify the downregulation. U6 was used as an internal control. **d**–**g** M1 macrophages were treated with PBS or Hyp/Exo in the presence of BLZ945 or miR-21-silenced Hyp/Exo (ZipmiR-21). Vehicle and ZipmiR-Cont were used as controls for BLZ945 and ZipmiR-21, respectively. The levels of iNOS, Arg-1, P-Akt (Ser473), and P-Akt (Thr308) in the treated M1 macrophages were examined by immunoblotting analysis. GAPDH and Akt (pan) were used as loading controls (**d**). The percentage of CD86^+^ or CD206^+^ cells among F4/80^+^ cells in the treated M1 macrophages was quantified by using flow cytometry analysis (**e**). The concentrations of M-CSF (**f**) and IL-10 (**g**) in the CdM of the treated M1 macrophages were examined by using ELISA. *p < 0.05, **p < 0.01, and ***p < 0.001. All data are expressed as the mean ± SD of *n* = 3

As our results above demonstrated that Hyp/Exo had a greater impact on M2 polarization than Nor/Exo, miRNA profiling was performed for Nor/Exo and Hyp/Exo to define the underlying molecular mechanisms of M2 polarization induced by exosomes from ASCs (Figure V in the online-only Data Supplement). The profiling analysis identified 501 exosomal miRNAs with a ratio of Hyp/Exo to Nor/Exo greater than 2:1 (Fig. 6a). Based on an extensive review of reported M2 polarization-regulating miRNAs [12, 27–31], seven of the 501 miRNAs were considered to promote M2 polarization. The seven miRNAs, miR-21, miR-223, Let-7c, miR-146b, miR-33, miR-511-3p, and miR-181a-5p, were validated by RT-PCR analysis (Fig. 6b). Among these miRNAs, miR-21 showed the greatest increase in Hyp/Exo compared with Nor/Exo. Downregulation of miR-21 in Hyp/Exo was achieved by transducing Lenti/ZipmiR-21 into donor hypoxic ASCs (Fig. 6c). When M1 macrophages were treated with miR-21-knockdown Hyp/Exo (ZipmiR-21), the extent of the decrease in iNOS expression and the increase in Arg-1 expression and Akt (Ser473 and Th308) phosphorylation by the control Hyp/Exo (ZipmiR-Cont) were reduced (Fig. 6d). The effects of Hyp/Exo on downregulating the percentage of CD86-positive macrophages and upregulating the percentage of CD206-positive macrophages were also reduced by knockdown of miR-21 in Hyp/Exo (Fig. 6e). The concentrations of M-CSF (Fig. 6f) and IL-10 (Fig. 6g) in the CdM of M1 macrophages treated with miR-21-knockdown Hyp/Exo were much lower than those in the group treated with the control Hyp/Exo. When BLZ945 was used along with Hyp/Exo, similar effects to those of miR-21-knockdown Hyp/Exo were observed (Fig. 6d–g). These results suggest that miR-21 in Hyp/Exo from ASCs plays an important role in exosome-induced M2 polarization by regulating Akt phosphorylation.

## Discussion

As a critical component of innate immunity, macrophages regulate the balance between inflammatory activity and tissue repair [32]. The polarization of macrophages to the M2 phenotype promotes angiogenesis and is a potential therapeutic approach to ischemic diseases [10–12]. Our previous studies have shown that exosomes from ASCs directly target vascular endothelial cells to promote angiogenesis [6]. Here, we demonstrate that exosomes enhance proangiogenic effects by targeting macrophages to polarize the M1 to M2 phenotype.

ASC-derived exosomes were recently implied to reduce inflammation in white adipose tissue through reprogramming adipose macrophages from the M1 to M2 subtype [17]. We provide evidence that exosomes from ASCs, particularly from hypoxic ASCs, induce mouse M1 macrophage skewing to an M2-like phenotype partially by activating CSF-1R. Exosomes from hypoxia-treated human ASCs enhance endothelial cell tube formation [33]. Hypoxia incubation is a mimic of the ischemic circumstance of cells. ASCs secrete more exosomes under ischemia, which implies a release of more signals requiring more blood or oxygen supply. We further revealed that exosomes from hypoxic human ASCs induce human monocyte-derived M1 macrophages to be polarized to an M2-like phenotype. Compared with M1 macrophages, our data show that M2-like macrophages polarized by exosomes from ASCs secrete more angiogenic factors and that CdM from M2-like macrophages promotes endothelial cell proliferation, migration, and tube formation. We and other laboratories have reported that exosomes from ASCs promote angiogenesis [4–6, 33]. However, the possible synergistic effects between exogenous proangiogenic exosomes and endogenous proangiogenic M2 macrophages are poorly understood. Our findings demonstrate that exosomes from hypoxic ASCs do not increase the total number of macrophages but do increase the percentage of the M2 population while enhancing arteriogenesis, angiogenesis, and blood perfusion in mouse angiogenic models. The administration of the CSF-1R inhibitor BLZ945 affects the efficacy of exosomes in the ischemic hindlimb. A recent study demonstrated that exosomes from bone marrow-derived MSCs attenuate myocardial ischemia-reperfusion injury through macrophage polarization, which is consistent with our observations [34].

miRNAs in exosomes play an essential role in mediating cell-cell communication. Based on an extensive review of the literature, two dozen miRNAs were found to induce macrophage M2 polarization [34, 35]. Among them, seven miRNAs were enriched in Hyp/Exo from ASCs, namely, miR-21, miR-223, Let-7c, miR-146b, miR-181a, miR-33, and miR-511-3p, according to our miRNA array results. miR-21 was validated as having the greatest increase and was shown to contribute to the induction of macrophage M2 polarization by Hyp/Exo from ASCs. According to the Ingenuity Pathway Analysis prediction, 80% of miR-21 targets are associated with macrophage polarization toward an inflammatory phenotype, whereas only 10% are associated with the M2 phenotype. Inhibition of miR-21 in macrophages has been demonstrated to reduce macrophage M2 polarization [12, 27–31]. Exosomes from miR-21-downregulated cancer cells have also been observed to indirectly mitigate macrophage M2 polarization [36]. However, in the mouse macrophage cell line RAW264.7, the miR-21 mimic attenuates M2 polarization, while the miR-21 inhibitor augments M2 polarization [35]. This opposite observation may be caused by the immortalization and transformation of the cell line.

miR-21 targets phosphatase and tensin homolog (PTEN), which inhibits the phosphoinositide 3-kinase (PI3K)/Akt pathway [37]. As one of the most extensively studied signaling pathways, the PI3K/Akt pathway is involved in almost every aspect of macrophage activation [38, 39]. An Akt allosteric inhibitor suppresses Akt phosphorylation and M2 polarization [40]. Similarly, our results show that Hyp/Exo with downregulated miR-21 or together with the CSF-1 inhibitor BLZ945 decreases Akt phosphorylation, M2 polarization, and cytokine secretion, including M-CSF. miR-21 is reported to elevate CSF-1 secretion through PTEN downregulation and Akt phosphorylation in breast tumors [41]. CSF-1 binds to CSF-1R, reinforces PI3K/Akt activation, and promotes M2 polarization, which can be blocked by a CSF-1R inhibitor [20, 42]. These observations imply that CSF-1 autocrine/paracrine signaling is critical in exosomal miR-21-induced PI3K/Akt pathway activation and M2 polarization as a positive feedback (Figure VI in the online-only Data Supplement). However, miR-21 is unlikely to be the only factor responsible for exosomes from hypoxic ASCs inducing M2 polarization. Other miRNAs enriched in Hyp/Exo, either reported to be macrophage polarization-related or not, may also have the capacity to promote M2 polarization. In addition, exosomes from ASCs carry a variety of proteins that are biologically active and can trigger functional responses in target cells [20]. Indeed, exosomal proteins from tumors have been recently reported to modulate M2 macrophage polarization [43]. Additional studies regarding the effects of proteins in exosomes from hypoxic ASCs on M2 polarization may be needed to further elucidate the underlying molecular mechanisms.

## Conclusion

In summary, our results reveal that exosomes derived from ASCs induce M1 macrophages from mice or humans to be polarized to a proangiogenic M2-like phenotype. Hypoxic preconditioning in ASCs enhances the quantity of secreted exosomes as well as the ability of secreted exosomes to promote angiogenesis and M2 polarization, which can be reduced by a CSF-1R inhibitor. Exosomal delivery of miR-21 contributes to M2-like polarization and the secretion of proangiogenic cytokines, including CSF-1. The PI3K/Akt pathway may mediate miR-21 delivery-induced CSF-1R activation and M2 polarization. This study uncovers a novel underlying mechanism of exosomes derived from hypoxic ASCs as a therapeutic strategy for ischemic diseases and expands our understanding of the role of the immune system in the response to therapeutic angiogenesis. The effect of exosomes on regulating the polarization of macrophages may also imply their broader application for certain immune diseases by modulating immune responses and promoting tissue repair.

## Supplementary information

**Additional file 1.**

## Data Availability

The data that support the findings of this study are available from the corresponding author upon reasonable request.

## Abbreviations

Arg-1: Arginase-1
ASC: Adipose-derived stem cell
bFGF: Basic fibroblast growth factor
CFSE: Carboxyfluorescein succinimidyl ester
CMVEC: Cardiac microvascular endothelial cell
CSF-1: Colony stimulating factor 1
CSF-1R: Colony stimulating factor 1 receptor
GM-CSF: Granulocyte-macrophage-colony stimulating factor
HLI: Hindlimb ischemia
Hyp/Exo: Exosomes from hypoxic adipose-derived stem cells
IL: Interleukin
iNOS: Inducible nitric oxide synthase
MSC: Mesenchymal stem cell
miRNA/miR: MicroRNA
M-CSF: Macrophage-colony stimulating factor
Nor/Exo: Exosomes from normoxic adipose-derived stem cells
PTEN: Phosphatase and tensin homolog
VEGF: Vascular endothelial growth factor
ZipmiR-21: Lenti/ZipmiR-21
ZipmiR-Cont: Lenti/ZipmiR-Control
