# Supplementary material for: Macrophage M2 polarization induced by exosomes from adipose-derived stem cells contributes to the exosomal proangiogenic effect on mouse ischemic hindlimb
Source: Stem Cell Res Ther. 2020 Apr 22;11:162. doi: 10.1186/s13287-020-01669-9 (PMC7178595; doi:10.1186/s13287-020-01669-9)
Supplement: Supplementary file 1 — Additional file 1. [file 13287_2020_1669_MOESM1_ESM.pdf]

SUPPLEMENTAL MATERIALS

Materials and Methods

Exosome isolation from human ASCs

Human ASC was purchased from Thermo Fisher Scientific and was maintained in MesenPRO RS medium (Thermo Fisher Scientific) at 37°C in a humidified 5% CO_2 incubator. The medium was replaced every 2-3 days, and passages 4-6 were used for all experiments. The methods of hypoxia precondition of the cells and isolation of exosomes from the cell culture medium were the same as described in the main text.

Macrophage differentiation from human CD14$^+$ monocyte

Human CD14$^+$ monocyte was purchased from the PromoCell (Heidelberg, Germany). M1 polarization was induced by incubating the monocytes in Macrophage-SFM supplemented with 30 ng/ml GM-CSF and 30 ng/ml IL-6 (R&D Systems) for 7 days. M2 polarization was induced by incubating the monocytes in Macrophage-SFM supplemented with 30 ng/ml M-CSF for 7 days. The induced M2 macrophage was used as a positive control in this study.

Flow cytometry and immunoblotting analysis for human macrophages

Human M1 macrophages were treated with 30 μg/ml Hyp/Exo from human ASCs in Macrophage-SFM for indicated days. FITC-anti-CD163 and PE-anti-CD80 antibodies (BioLegend) were used for flow cytometry analysis. Primary anti-STAT1 (14994S, Cell Signaling) and anti-STAT3 (9139S, Cell Signaling) antibodies were used for immunoblotting analysis.

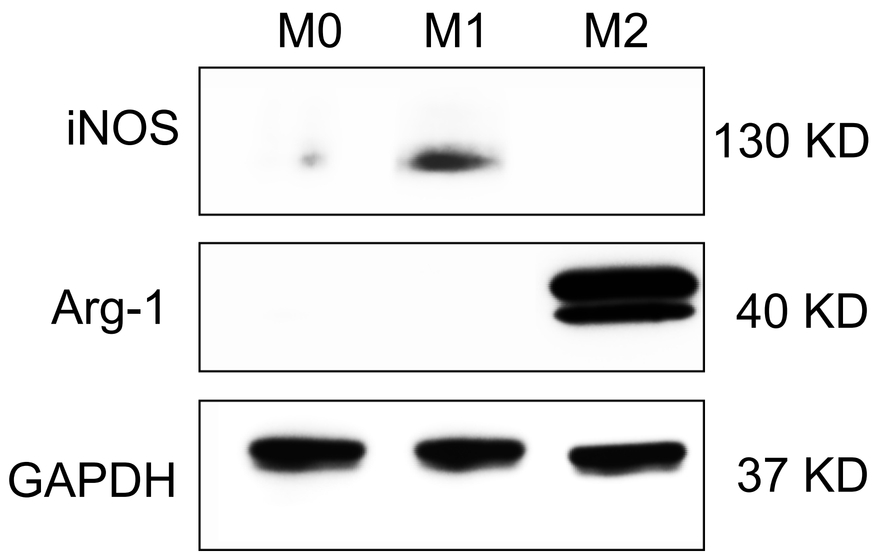

Figure I. Identification of macrophage polarization. M0 macrophages were treated with indicated cytokines for 48 hours as described in the Materials and Methods to be polarized to M1 or M2 macrophages. Macrophage intracellular markers, iNOS for M1 and Arg-1 for M2, were examined by using immunoblotting analysis. GAPDH was used as a loading control (n = 3).

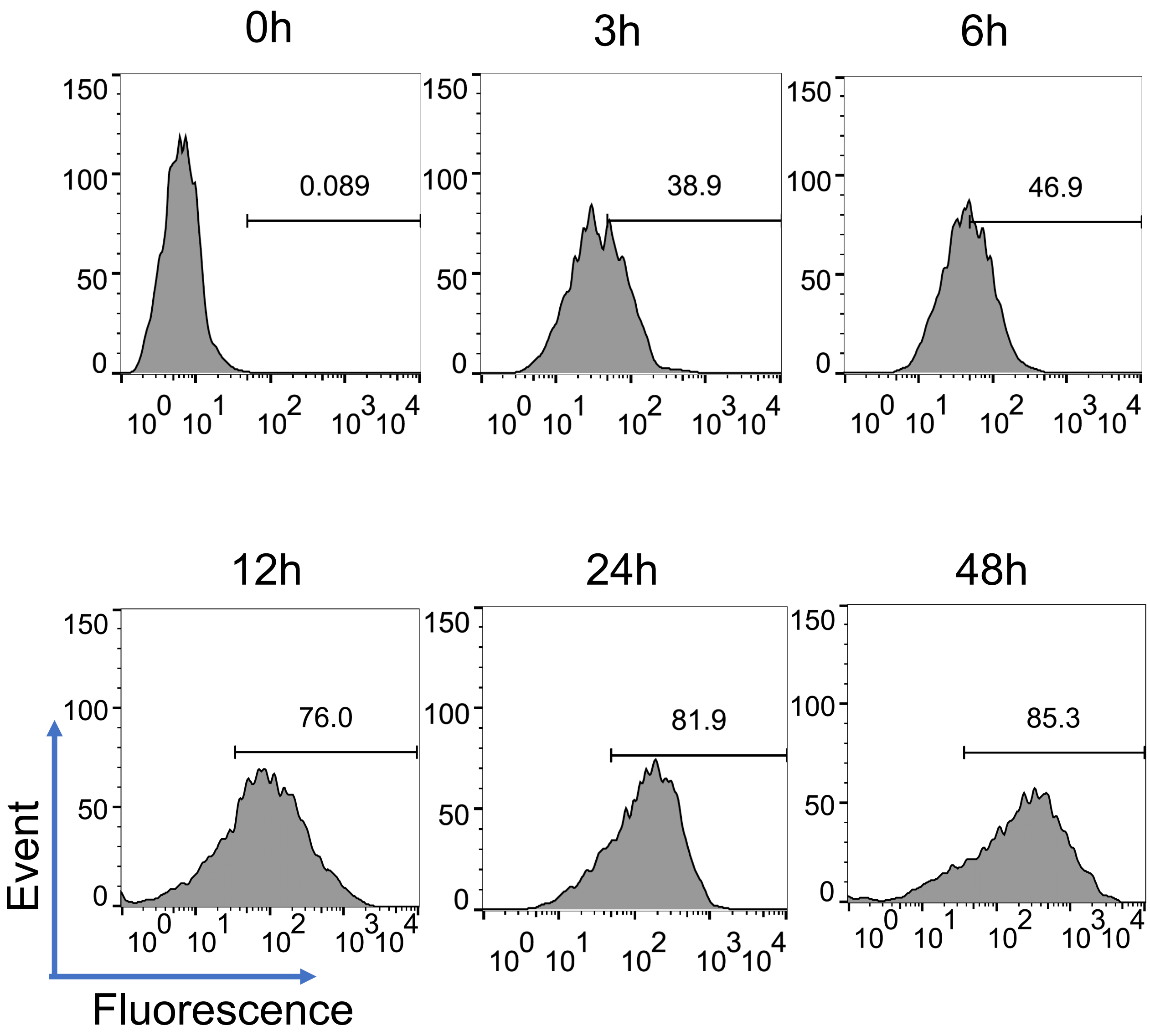

Figure II. Uptake of exosomes into M1 macrophages. M1 macrophages were incubated with Exo-Green labeled exosomes at a concentration of 30 μg/ml for indicated times. The percentage of fluorescence positive macrophages were measured by using flow cytometry (n = 3).

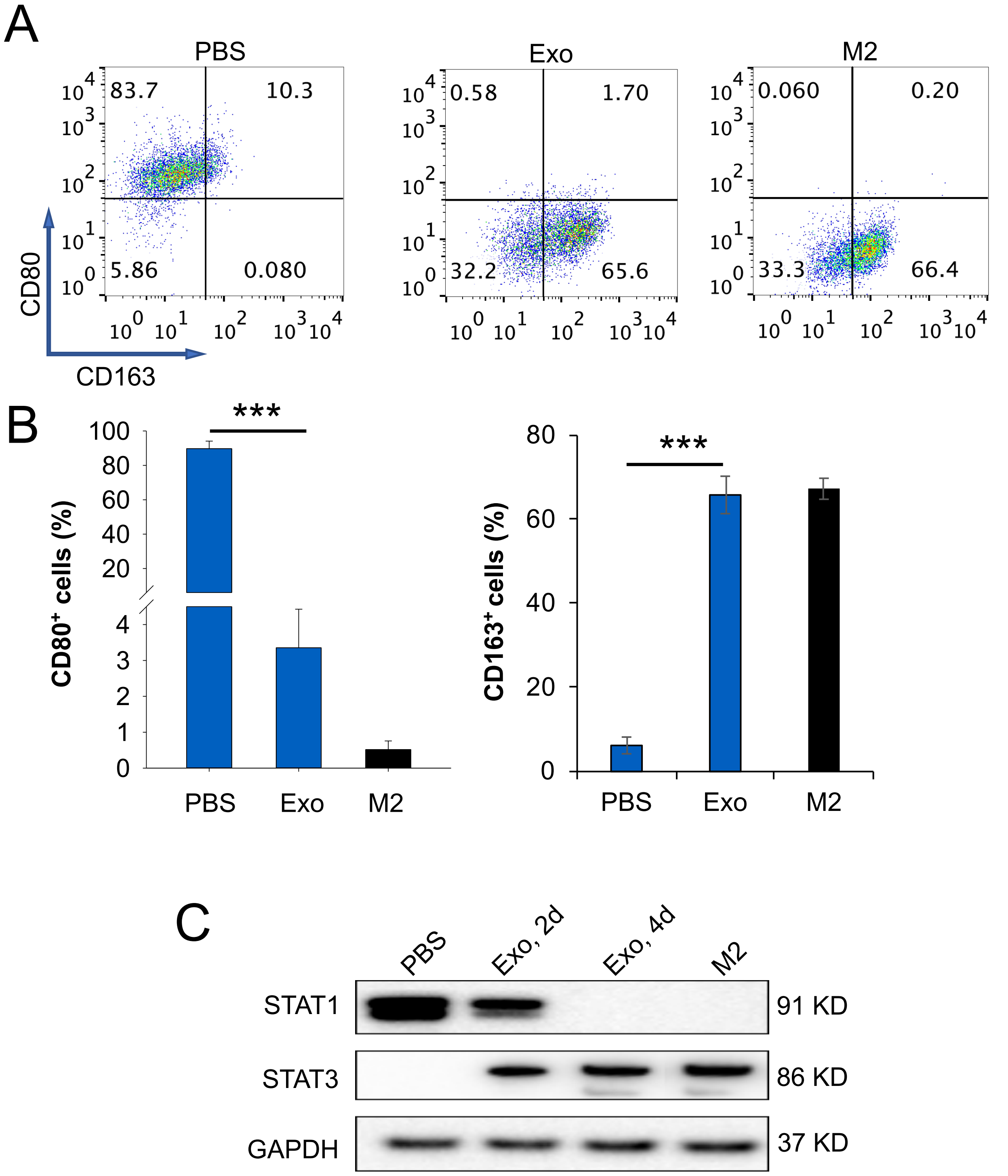

Figure III. Human M1 macrophages are polarized to M2-like phenotype by Hyp/Exo from human ASCs. Human M1 macrophages were treated with Hyp/Exo from human ASCs for 4 days. PBS was used as a negative control. M2 was used as a positive control. **A** and **B**, Human macrophage surface markers, CD80 for M1 and CD163 for M2, were examined by using flow cytometry (A) (n = 3). (B) is the statistical results of panel A. **C**, Time course of human macrophage intracellular markers, STAT1 for M1 and STAT3 for M2, were examined by using immunoblotting. GAPDH was used as a loading control (n = 3). *** p<0.001.

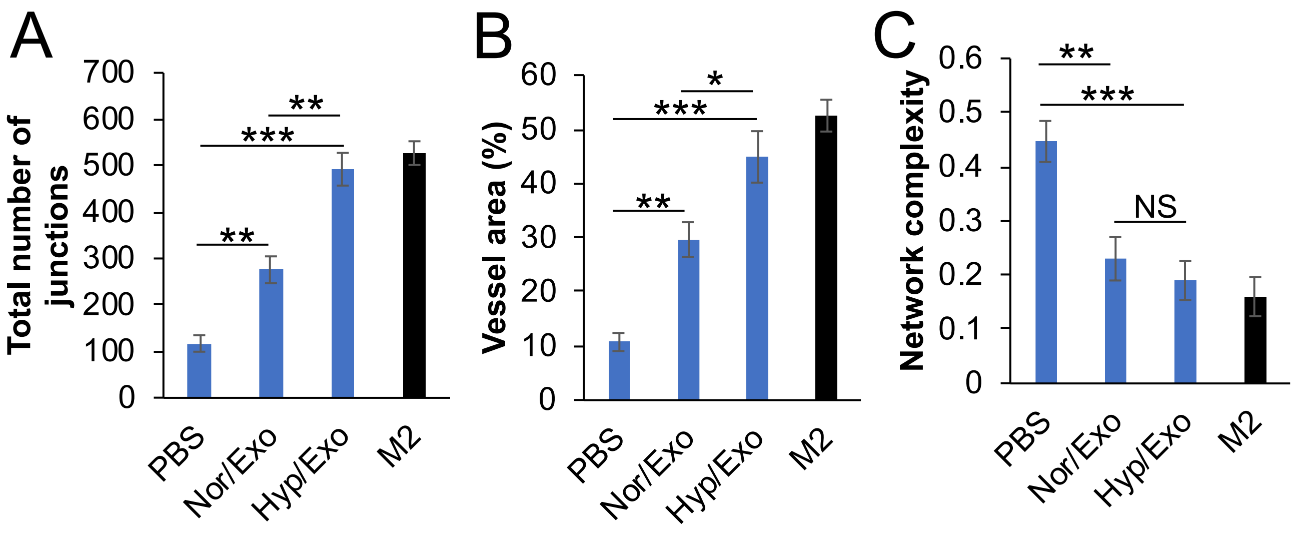

Figure IV. The conditioned medium (CdM) from the exosome-polarized M2-like macrophages promotes tube formation in CMVEC. M1 macrophages were treated with 30 μg/ml of Nor/Exo or Hyp/Exo for 48 hours. The cells were then washed and incubated in fresh Macrophage-SFM for another 48h. The culture medium was collected and used as CdM. CMVECs were treated with the various CdM from macrophages. PBS was used as a negative control. Tube formation assay of the CMVECs were performed. The total number of junctions (A), vessel area (B), and network complexing (C) were measured. The CdM from M2 was used as a positive control. **P*<0.05, ***P*<0.01 and ****P*<0.001. NS, not significant. Data are expressed as mean ± SD of n = 3.

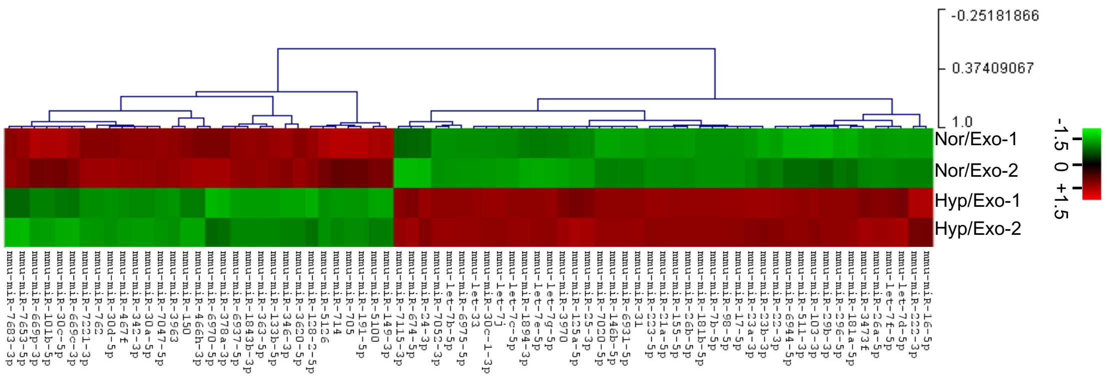

Figure V. Profiling of miRNAs in Nor/Exo and Hyp/Exo. Heat map showed the miRNAs only with the difference between Nor/Exo and Hyp/Exo (met both conditions: $p < 0.1$ and fold change > 2). Hierarchical clustering displayed on the axis illustrated miRNA cluster relationships (n = 2).

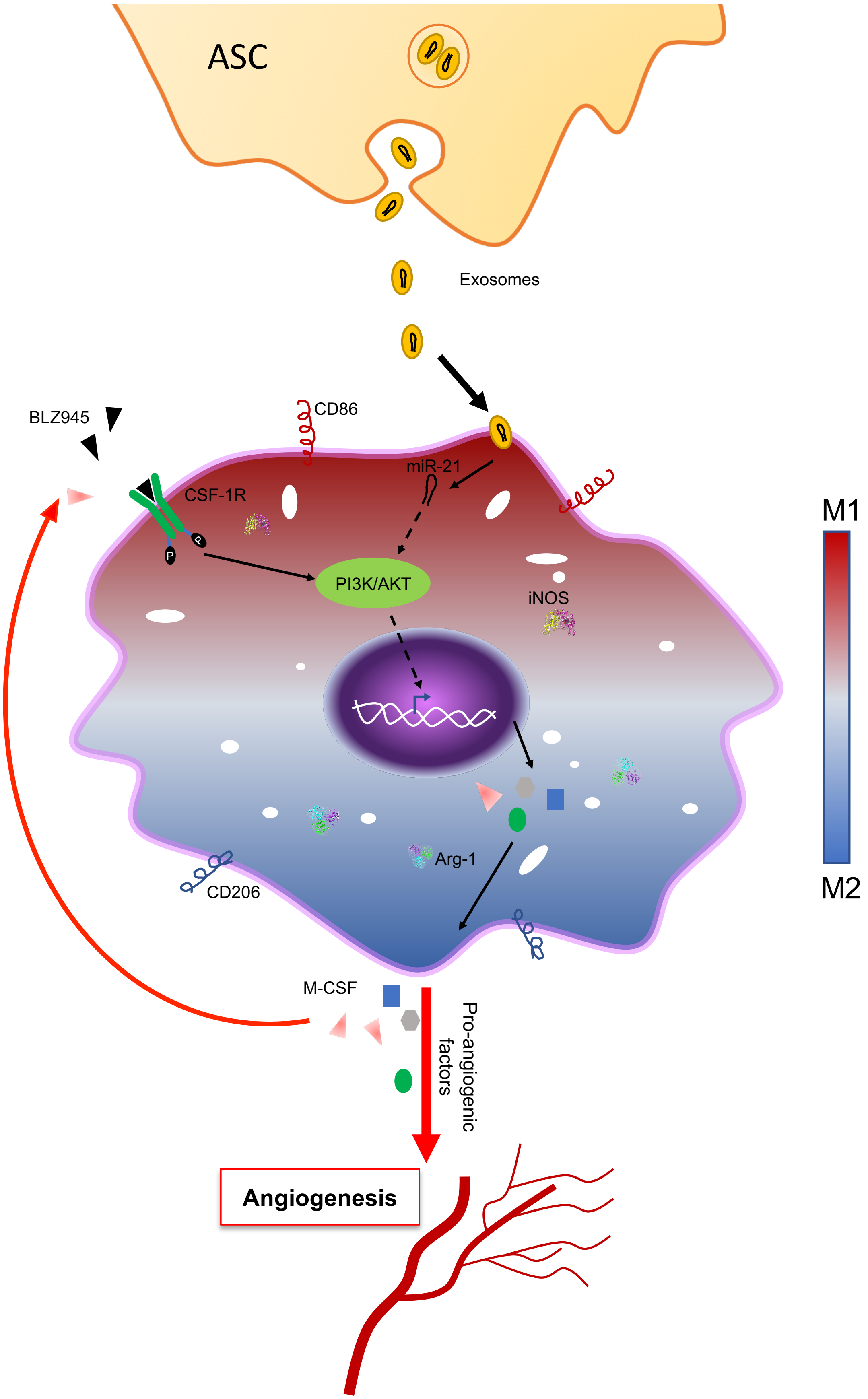

ASC
Exosomes
BLZ945
CD86
miR-21
CSF-1R
P
P
PI3K/AKT
iNOS
Arg-1
CD206
M1
M2
M-CSF
Pro-angiogenic factors
Angiogenesis
